# Burden of disease among patients with prevalent Crohn’s disease: results from a large German sickness fund

**DOI:** 10.1007/s00384-023-04368-y

**Published:** 2023-03-20

**Authors:** Evi Zhuleku, Beatriz Antolin-Fontes, Andras Borsi, Riikka Nissinen, Ivana Bravatà, Jennifer Norma Barthelmes, Jennifer Lee, Alun Passey, Daniel Wirth, Ulf Maywald, Bernd Bokemeyer, Thomas Wilke, Marco Ghiani

**Affiliations:** 1 Cytel Inc, Berlin, Germany; 2 Janssen-Cilag Ltd, High Wycombe, UK; 3 Janssen-Cilag Oy, Espoo, Finland; 4 Janssen-Cilag S.P.A, Milan, Italy; 5 Janssen-Cilag GmbH, Neuss, Germany; 6 Healthsystemcreativity, Hamburg, Germany; 7 Interdisciplinary Crohn Colitis Centre Minden, Minden, Germany; 8 Institut Für Pharmakoökonomie Und Arzneimittellogistik (IPAM) E.V, Wismar, Germany

**Keywords:** Crohn’s disease, Real-world-evidence (RWE), Active disease, Steroid-dependency, Treatment guidelines

## Abstract

**Purpose:**

The aim of this study was to investigate the burden of disease among a real-world cohort of patients with prevalent Crohn’s disease (CD) in Germany.

**Methods:**

We conducted a retrospective cohort analysis using administrative claims data from the German AOK PLUS health insurance fund. Continuously insured patients with a CD diagnosis between 01 October 2014 and 31 December 2018 were selected and followed for at least 12 months or longer until death or end of data availability on 31 December 2019. Medication use (biologics, immunosuppressants (IMS), steroids, 5-aminosalicylic acid) was assessed sequentially in the follow-up period. Among patients with no IMS or biologics (advanced therapy), we investigated indicators of active disease and corticosteroid use.

**Results:**

Overall, 9284 prevalent CD patients were identified. Within the study period, 14.7% of CD patients were treated with biologics and 11.6% received IMS. Approximately 47% of all prevalent CD patients had mild disease, defined as no advanced therapy and signs of disease activity. Of 6836 (73.6%) patients who did not receive advanced therapy in the follow-up period, 36.3% showed signs of active disease; 40.1% used corticosteroids (including oral budesonide), with 9.9% exhibiting steroid dependency (≥ 1 prescription every 3 months for at least 12 months) in the available follow-up.

**Conclusions:**

This study suggests that there remains a large burden of disease among patients who do not receive IMS or biologics in the real world in Germany. A revision of treatment algorithms of patients in this setting according to the latest guidelines may improve patient outcomes.

## Introduction

Crohn’s disease (CD) belongs to the class of inflammatory bowel diseases (IBD) presenting at any age and resulting in significant morbidity and decreased quality of life [1]. Over time, many patients develop complications, with approximately half of the CD population requiring surgery in the 10 years after initial diagnosis [2, 3].

The German Society for Gastroenterology, Digestive and Metabolic Diseases (DGVS) recommends oral budesonide or steroids for inducing clinical remission among patients with mild-to-moderate CD, remarking that the therapeutic efficacy of 5-aminosylicilic acid (5-ASA) remains limited based on available evidence [4]. In patients with steroid dependency, immunosuppressants (IMS) can be used. Treatment guidelines, including the latest revisions from the European Crohn’s and Colitis Organisation (ECCO), indicate the use of biologics, such as anti-tumor necrosis factors (anti-TNFs) including infliximab (IFX) and adalimumab (ADA), agents targeting leukocyte trafficking, namely vedolizumab (VDZ), or the anti-p40 (anti-Interleukin-12/23) antibody, ustekinumab (UST) for induction and maintenance of remission in patients with moderate-to-severely active CD with inadequate response to therapy with steroids and/or IMS [3, 4].

With the emergence of new therapeutic options and evolving guidelines, we evaluated the treatment and burden of disease among a real-world cohort of patients with CD in Germany based on claims data. Upon identification of a subpopulation of patients who were not treated with biologics or IMS (advanced therapy), we further evaluated indicators of active disease and steroid dependency to assess any unmet therapeutic needs.

## Materials and methods

This study is a retrospective analysis of anonymized claims data from the AOK PLUS statutory health insurance fund in Germany. AOK PLUS captures data on approximately 3.4 million insured individuals in the regions of Saxony and Thuringia. Patient information on demographics (age, sex), inpatient care (hospitalizations, procedures), and outpatient care (diagnoses, procedures, prescriptions) was extracted.

We selected a cohort of prevalent adult patients with at least one inpatient and/or two outpatient diagnoses of CD (ICD-10-GM: K50.-) between 01 October 2014 and 31 December 2018, with no subsequent diagnosis of ulcerative colitis (UC, ICD-10-GM: K51.-) after the first CD diagnosis in the inclusion period (prevalent CD). Patients without continuous insurance for at least 12 months after the first CD diagnosis in the inclusion period were excluded from the final sample. Patients were followed for at least 12 months after the first observable CD diagnosis or longer until death or end of data availability on 31 December 2019. We then assessed the use of medications including biologics, IMS, corticosteroids, and 5-aminosalicylic acid (5-ASA) in the entire available follow-up period.

Moreover, from the overall prevalent CD population, we identified a subgroup of patients without advanced treatment after the first observed CD diagnosis in the inclusion period. Advanced therapies included any prescription of IMS, including azathioprine (ATC: L04AX01), mercaptopurine (ATC: L01BB02), cyclosporin (ATC: L04AD01), tacrolimus (ATC: L04AD02), methotrexate (ATC: L01BA01, L04AX03), or leflunomide (ATC: L04AA13), and biologics, including IFX (ATC: L04AB02), ADA (ATC: L04AB04), VDZ (ATC: L04AA33), or UST (L04AC05). Patients without advanced therapy (i.e., biologics and IMS) were analyzed for claims-based indicators of active disease, defined in this study as the presence of at least two prescriptions of corticosteroids including oral budesonide within 12 months, at least one CD-related surgery (Operation and Procedure Codes (OPS): 5–45, 5–46, 5–48, 5–49), or at least one CD-related hospitalization (main/primary diagnosis ICD-10-GM: K50.-) with a length of stay > 7 days based on prior literature and clinical input (proxy) [5]. Corticosteroid use among this subpopulation was evaluated and the number of patients with steroid dependency, defined as at least one corticosteroid prescription every three months for at least 12 months was reported.

## Results

Between 01 October 2014 and 31 December 2018, we identified 9284 prevalent adult CD patients with a follow-up of at least 12 months. Among these prevalent CD patients, 1367 (14.7%) patients received biologics (+ / − IMS), of which 1254 (13.5%) received anti-TNFs. Among the remaining patients without anti-TNF therapy, an additional 113 (1.2%) patients received VDZ (77/113) and/or UST (51/113) in the follow-up. Moreover, an additional 1081 (11.6%) patients were treated with IMS only in the entire available follow-up period, of which 1028/1081 (95.1%) received at least one prescription of azathioprine. The remaining 6836 (73.6%) patients without advanced therapy (IMS or biologics) received 5-ASA, other non-advanced therapies for CD (including corticosteroids), or no therapy.

The subpopulation of patients without advanced therapy in the follow-up period exhibited a mean age of 52.0 years at the date of first observable CD diagnosis, and 61.3% were female (Table 1). The median length of follow-up was 4.4 years. Overall, 4356 patients (63.7% of those without advanced therapy; 46.9% overall) did not present with active disease indicators. Active disease indicators were present for 36.3% of patients in this subpopulation, with 28.3%, 11.0%, and 5.6% having at least two prescriptions of steroids within 12 months, at least one CD-related surgery, or at least one CD-related hospitalization with (> 7 days), respectively (Table 1).

**Table 1:** Patient characteristics and therapy use among patients without advanced therapy in the follow-up period (≥ 12 months)

Patients without advanced therapy (biologics/IMS)	*N* = 6836
**Demographics**	
Age at CD diagnosis, mean years (SD)	51.9 (18.3)
**Age groups**	
**18–44 years,** ***n*** (%)	2556 (37.4)
**45–64 years,** ***n*** (%)	2504 (36.6)
**≥ 65 years,** ***n*** (%)	1776 (26.0)
Female, ***n*** (%)	4189 (61.3)
Length of follow-up, median years (IQR)	4.4 (2.7–5.2)
**Active disease**	
Patients without active disease, ***n*** (%)	4356 (63.7)
Patients with active disease (at least 1 of the following), ***n*** (%)	2480 (36.3)
At least 2 prescriptions of steroids within 12 months, ***n*** (%)	1937 (28.3)
At least 1 CD-related surgery, ***n*** (%)	749 (11.0)
At least 1 CD-related hospitalization, ***n*** (%)	383 (5.6)
**Corticosteroids usage**	
Any corticosteroids (incl. oral budesonide), ***n*** (%)	2784 (40.7)
Budesonide (oral), ***n*** (%)	1337 (19.6)
Other steroids, e.g., prednisolone/prednisone (oral), ***n*** (%)	1981 (29.0)
Budesonide and other steroids (oral), ***n*** (%)	534 (7.8)
Number of corticosteroid prescriptions, mean (SD) | median (IQR)	6.4 (8.4) | 3 (1–8)
Steroid dependency, ***n*** (%)	676 (9.9)

*CD* Crohn’s disease, *IMS* Immunosuppressants, *IQR* Interquartile range, *SD* Standard deviation

We further investigated the use of corticosteroids in the follow-up period among patients without advanced therapy. High proportions of steroid use were observed, with 2784 (40.7%) of CD patients without advanced therapy receiving at least one prescription including oral budesonide. Among these patients, the mean number of corticosteroid prescriptions during the whole follow-up period was 6.4 (Table 1). Approximately a third of patients (29.0%) were treated with oral corticosteroids such as prednisone/prednisolone (excluding budesonide), whereas 19.6% received oral budesonide. Specifically, among 17,765 total prescriptions in the follow-up period, oral budesonide was the most frequently prescribed (56.0% of prescriptions), followed by prednisolone (32.8% of prescriptions). A proportion of patients were heavily treated with corticosteroids, with 9.9% (676/6838) defined as steroid dependent (Table 1).

## Discussion

This study evaluated real-world therapy use among a prevalent cohort of CD patients treated in Germany between 2014 and 2019. Analyses of real-world patterns such as those presented by this research are important in identifying unmet needs.

Our study shows that approximately 13.5% of prevalent patients were treated with biologics after the first observable CD diagnosis in the study period, in line with previous reports in studies assessing similar time frames, with the literature based on other European settings generally showing an increase in the use of biologics over time (15.0% to 18.7% from 2011–2017 in Catalonia [6], 8.9% to 14.5% from 2011–2018 in Denmark [7], 21% to 33% from 2011 to 2016 in Norway within the first year of diagnosis [8]). In the same Spanish study, the use of 5-ASA and corticosteroids decreased over time from 2011 to 2017 from 28.8% to 17.1% and 15.8% to 13.7%, respectively [6]. In the present study, approximately half of the prevalent CD population (46.9%) were treated with only 5-ASA, other non-advanced therapy, or no therapy and had no indications of active disease. This suggests that a large proportion of prevalent CD patients in our cohort presented with a mild course of disease, a finding which is directly relevant for the classification and selection of an appropriate therapeutic approach for these patients. In a retrospective multicenter study in Germany between 2007 and 2010, Kruis et al. showed nearly a third of CD patients had a mild course of disease [9].

Among patients with prevalent CD, we further identified and described a subpopulation who were not treated with biologic agents or IMS (advanced therapy) in the available follow-up period (73.6%). However, 36% of patients within this subpopulation showed signs of active disease and 41% used corticosteroids in the follow-up period. Active disease indicators and corticosteroid use in this setting may suggest a need for a change in therapy for improved disease management [5]. Treatment of these patients appears to deviate from the latest treatment guidelines [3, 4]. For instance, 5-ASA, commonly used to treat a mild CD flare, is not recommended by ECCO or DGVS due to heterogenous data and low efficacy. In alignment, according to a 2015 web-based survey of 175 German gastroenterologists, the use of 5-ASA in clinical practice showed a tendency to diverge from guidelines [10]. A significant proportion of patients in our population were also treated longer term with steroids, deviating from the goal of achieving steroid-free remission as outlined by CD guidelines [3, 4]. Furthermore, long-term corticosteroid use is associated with further complications, including bone loss, metabolic complications, significant infections, among other serious risks [11]. Treatment guidelines recommend that patients with steroid-dependent CD, detected in 9.9% of our patient population, should receive IMS or biologics to manage their disease. Overall, the significant proportion of patients with active disease and corticosteroid use suggests that real-world practice in this setting may not be closely aligned to updated guidelines.

While secondary diagnoses were not evaluated in this study, it is important to highlight that patients with active disease often present with a disease course that results in fibrotic stenosis [12]. Among such patients, advanced medications such as biologics would not be beneficial, and patients should undergo surgery to remove stenosis. In this case, corticosteroids may be given during flare-ups in order to narrow the stenosis due to inflammation. As such, a better understanding of concomitant diagnoses such as chronic fibrotic stenosis is needed to further contextualize the high corticosteroid use observed in this study. Specifically, a study using standardized hospital discharge data from 2010 to 2017 in Germany showed that despite the introduction of novel biologics, the number of patients with CD requiring surgery remained stable, with patients increasingly hospitalized with stenosis and malnutrition over time [13]. Based on the results from this short study, we highlight the need for future research in the area, including a more comprehensive description of secondary conditions and a comparison of the evolution of therapy use, surgeries, and hospitalizations over time. Moreover, a contextualization via the description of active disease indicators and corticosteroid use among patients with advanced therapy would serve beneficial.

Associated with the nature of the data, there are some limitations to this analysis. The dataset captures patients in the regions of Saxony/Thuringia and may be subject to a degree of selection bias. However, healthcare regulations are considered uniform across Germany, minimizing bias and conferring representativity. Furthermore, clinical information on disease severity, active disease, and disease manifestation (Montreal classification) was not available and was supplemented by claims data-based proxies. Further information on demographics such as race, ethnicity, income, and education was not available. While claims data captures all prescriptions filled, compliance cannot be directly ascertained. Finally, claims data is captured in the context of daily clinical practice and may be associated with a small degree of miscoding, particularly in the outpatient setting. Moreover, this study has several strengths. Using a large claims database, we made use of hospitalization, procedure, and prescription data to describe the study populations. Definitions for active disease and steroid dependency were based on prior studies and clinical practice [5]. The database allowed for the inclusion of all CD patients with any disease course, thus providing a representative picture of the treatment of CD in Germany.

## Conclusions

The results suggest that approximately half of the CD patients included in this study had a relatively mild course of CD. However, there may persist a great burden of disease among the remaining proportion of CD patients who did not receive immunosuppressants or biologics although showed signs of active disease in the real-world in Germany. Among these patients, a significant proportion used corticosteroids in the follow-up period despite treatment goals. To reduce the burden and improve outcomes in this cohort of patients, a revision of treatment strategies in clinical practice according to the latest treatment guidelines may be beneficial.

## Data Availability

Patient-level data used for this study cannot be made publicly available in accordance with the local laws and policies of the participating institutions (AOK PLUS and IPAM).

## References

[1] Torres J (2017). Crohn’s disease. Lancet.

[2] Peyrin-Biroulet L (2010). The natural history of adult Crohn’s disease in population-based cohorts. Am J Gastroenterol.

[3] Torres J (2020). ECCO guidelines on therapeutics in Crohn’s disease: medical treatment. J Crohns Colitis.

[4] Sturm A (2022). Updated S3 guideline “Diagnosis and therapy of Morbus Crohn” of the German Society for Gastroenterology, Digestive and metabolic diseases* (DGVS)*. Z Gastroenterol.

[5] Bokemeyer B (2020). Indicators of active disease and steroid dependency in patients with inflammatory bowel diseases not treated with biologics in a German real-world-setting. Int J Colorectal Dis.

[6] Brunet E et al (2020) Time trends of Crohn’s disease in Catalonia from 2011 to 2017. Increasing use of biologics correlates with a reduced need for surgery. J Clin Med 9(9) 10.3390/jcm9092896 PMC7563515 32911630

[7] Zhao M (2022). Trends in the use of biologicals and their treatment outcomes among patients with inflammatory bowel diseases – a Danish nationwide cohort study. Aliment Pharmacol Ther.

[8] Anisdahl K et al (2019) P710 The use of first-line biologics in patients with Crohn’s disease in Norway from 2011 to 2016. J Crohn's and Colitis 13(Supplement_1): p. S475-S476

[9] Kruis W (2013). Predictive factors for an uncomplicated long-term course of Crohn’s disease: a retrospective analysis. J Crohns Colitis.

[10] Klag T, Stange EF, Wehkamp J (2015). Management of Crohn’s disease - are guidelines transferred to clinical practice?. United European Gastroenterol J.

[11] Rutgeerts PJ (2001). Review article: the limitations of corticosteroid therapy in Crohn’s disease. Aliment Pharmacol Ther.

[12] Yoo JH, Holubar S, Rieder F (2020). Fibrostenotic strictures in Crohn’s disease. Intest Res.

[13] Stöss C (2021). Crohn’s disease: a population-based study of surgery in the age of biological therapy. Int J Colorectal Dis.

